# Primary Care Involvement and Health Care Utilization Among Patients With End-Stage Kidney Disease

**DOI:** 10.1001/jamanetworkopen.2026.0807

**Published:** 2026-03-05

**Authors:** Kunal Bailoor, Richard A. Hirth, Paula Guro, Mary K. Oerline, John M. Hollingsworth, Vahakn B. Shahinian

**Affiliations:** 1 Division of Nephrology, Department of Internal Medicine, University of Michigan, Ann Arbor; 2 Department of Health Management and Policy, University of Michigan School of Public Health, Ann Arbor; 3 Department of Internal Medicine, University of Michigan, Ann Arbor; 4 Dow Division of Health Services Research, Department of Urology, University of Michigan, Ann Arbor; 5 Department of Urology, University of Florida College of Medicine, Gainesville; 6 Division of Nephrology, Department of Internal Medicine, University of Michigan, Ann Arbor

## Abstract

**Importance:**

Patients with end-stage kidney disease receiving dialysis have disproportionate rates of emergency department utilization and hospitalization. Models of care with primary care physicians may be able to reduce these events, although prior literature has not clearly demonstrated a benefit in the population of patients with end-stage kidney disease.

**Objective:**

To determine whether having a primary care practitioner is associated with lower risk of hospitalization and emergency department utilization in the population of patients with end-stage kidney disease.

**Design, Setting, and Participants:**

Retrospective, cross-sectional study of patients for whom Medicare Fee for Service was the primary payer with end-stage kidney disease in the contiguous US receiving dialysis between January 1, 2018, and December 31, 2019. Differential distance to a dialysis facility with a high level of primary care involvement was an instrumental variable. Data were analyzed from January 1, 2019 to December 31, 2019.

**Exposure:**

Presence or absence of primary care physician; instrument of differential distance defined as difference between distance to nearest high primary care–utilizing dialysis facility and distance to nearest dialysis facility of any type, using above or below median differential distance as instrumental variable.

**Main Outcomes and Measures:**

Any emergency department visit, emergency department visit not resulting in hospitalization, or hospitalization.

**Results:**

Among 181 520 patients (mean [SD] age, 62.6 [14.1]; 102 966 [56.7%] male; 71 953 [39.6%] Black, 27 215 [15.0%] Hispanic, and 97 661 [53.8%] White) with end-stage kidney disease receiving hemodialysis, patients estimated to have primary care involvement by our instrument had a lower estimated risk of any emergency department visit not resulting in hospitalization (51.2%; 95% CI, 49.7%-52.7%) relative to those not estimated to have primary care involvement (72.1%; 95% CI, 69.2%-74.9%; *P* < .001). There was a lower estimated risk of any emergency department visit in this population as well (69.4%; 95% CI, 68.1%-70.7% in those estimated to have primary care involvement vs 75.0%; 95% CI, 72.5%-77.6% in those not estimated to have primary care involvement). There was no significant difference in estimated risk of any hospitalization.

**Conclusions and Relevance:**

In this cross-sectional study of patients with end-stage kidney disease receiving dialysis, primary care physician involvement was associated with reduced emergency department utilization.

## Key Points

**Question:**

Is the presence of a primary care physician (PCP) associated with the likelihood of emergency department visits or hospitalization for patients with end-stage kidney disease receiving dialysis?

**Findings:**

In a cross-sectional study of a national cohort study of patients receiving dialysis using differential distance as the instrument, patients with a PCP had a statistically significant lower relative risk of emergency department visits not resulting in hospitalization relative to those without a PCP (51.2% with a PCP vs 72.1% without a PCP).

**Meaning:**

These findings suggest that increasing utilization of PCPs for patients with end-stage kidney disease receiving dialysis may help reduce emergency department utilization.

## Teaser

This cross-sectional study evaluates whether having a primary care practitioner is associated with lower risk of hospitalization and emergency department utilization in patients with end-stage kidney disease (ESKD) who receive dialysis.

## Introduction

Over half a million patients in the US are receiving dialysis for end-stage kidney disease as of 2020, a population that is projected to grow.^1^ This patient population is extremely ill, with Medicare spending 34.7 billion dollars on their care in 2021.^1^ Emergency department (ED) visits and hospitalizations represent a substantial proportion of this spending, with patients with end-stage kidney disease using the ED at 4 times the national mean rate for all Medicare beneficiaries.^2^ A major driver of emergency department visits^3,4^ are complications and exacerbations of chronic medical conditions such as type 2 diabetes and congestive heart failure, which are highly prevalent in this population.^5^ While in the general population these comorbid conditions are often managed by primary care physicians (PCPs), among patients with end-stage kidney disease receiving dialysis, nephrologists sometimes become responsible for managing these comorbidities.^6^

The evidence is mixed on whether the involvement of PCPs is beneficial in this population. On one hand, PCPs may be more comfortable managing nonkidney disease in this population,^7^ may be more accessible on an urgent basis, and may help ensure better preventive care in this population.^8^ On the other hand, nephrologists often assess patients more frequently and may be more comfortable with assessing complications that arise from hemodialysis.^6^ Some data on the role of PCPs may come from the recent Comprehensive End-Stage Renal Disease (ESRD) Care Model, a novel model of value-based care that was piloted from 2015 to 2021. This model included practitioners voluntarily clustered into ESRD Seamless Care Organizations (ESCOs), some of which included partnerships between hemodialysis facilities and PCPs.^9^ Analysis of the model showed participants had lower rates of hospitalization and readmission,^10^ which may have been driven in part by participation of PCPs in ESCOs. In addition to data from novel models of care, a prior experiment integrating primary care into 2 dialysis facilities showed significant improvement in health-related quality of life.^11^ Finally, in a prior cohort study evaluating the outcomes of presence of a PCP for patients with end-stage kidney disease, it was shown that patients with a PCP had more ED visits, hospitalizations, and worse survival than those without a PCP. The authors noted, however, that this analysis was confounded by the older age and greater burden of comorbidity among patients receiving hemodialysis with a PCP.^12^

The association between primary care involvement and important outcomes like ED utilization in the population of patients with end-stage kidney disease receiving hemodialysis remains unclear. We conducted a national, retrospective, cross-sectional study of patients receiving dialysis between January 1, 2018, and December 31, 2019, examining the association between primary care involvement and ED utilization and hospitalization in this population, using methods to address the confounding seen in prior studies.

## Methods

### Data Source

We used the US Renal Data System (USRDS) database^13^ to identify patients who were alive, receiving any modality of dialysis, and for whom Medicare was the primary payer. The USRDS was also used to identify unique nephrology practitioner IDs and nurse-to-patient ratios at dialysis facilities. Data for primary care visits, including evaluation and management (E and M) codes and specialty codes, data on dialysis claims linked to USRDS physician identifier, and data on primary diagnosis codes for ED visits were obtained from Medicare claims data. Zip code data required to calculate the distance between patient and facility zip codes were obtained from a permanent SAS database using a function that returned the geodetic distance in miles between the geographical centers of 2 US Postal Service zip codes. We used the Census’s American Community Survey to obtain information on educational attainment by county of patient residence, median income by zip code of patient residence, and rural vs urban indicator for patient’s zip code. Dialysis facility star ratings (a composite measure of dialysis facility quality) were obtained from the Center for Medicare & Medicaid Services (CMS) Dialysis Facility Compare program.

### Study Population

**Figure 1.**
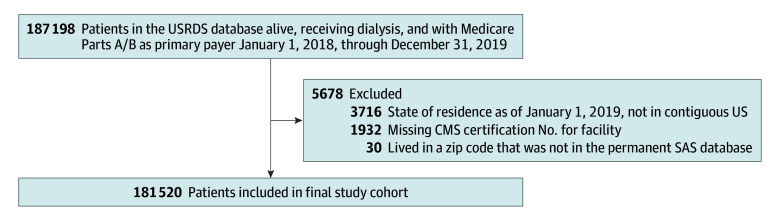
Flow Diagram of Inclusion and Exclusion Criteria for Cohort CMS, Centers for Medicare & Medicaid Services; USRDS, United States Renal Data System.

Our cohort consisted of patients in the USRDS database who were alive, receiving dialysis, and for whom Medicare Parts A and B were the primary payer between January 1, 2018, through December 31, 2019, initially consisting of 187 198 patients. We excluded patients with missing data, including missing CMS provider number for dialysis centers or missing zip code. We also excluded patients whose state of residence as of January 1, 2019, was not in the contiguous US, as we planned to use distance to dialysis facilities as part of our analysis, and these may be substantially different in noncontiguous states and territories. Figure 1 shows the inclusion and exclusion of patients from the study. The University of Michigan institutional review board deemed this study exempt and not regulated because data were deidentified. Informed consent was waived since this study used secondary coded private data and only restricted variables. This study followed the Strengthening the Reporting of Observational Studies in Epidemiology (STROBE) reporting guideline.^28^

### Study Variables

Our exposure of interest was whether patients receiving dialysis had a PCP involved in their care. This was defined by whether the patient had a relevant E and M code (eTable 1 in Supplement 1) from a practitioner with a Medicare primary care specialty code between January 1, 2018, and December 31, 2018. We used the following specialty codes: 01 (general practice), 08 (family practice), 11 (internal medicine), 37 (pediatric medicine), and 38 (geriatric medicine). We measured primary care visits in 2018 and our outcomes of interest in 2019 (ie, lagged), to avoid simply capturing new involvement of PCPs directly related to one of the outcome events.

Prior cohort studies directly comparing patients receiving dialysis with PCPs with those without have been confounded by unequal distributions of age and comorbidity.^12^ To address this confounding, we additionally planned to use an instrumental variable approach, specifically differential distance to a dialysis facility that had high primary care utilization. We defined our instrument in 2 steps. First, we expected to find substantial variation in primary care utilization by dialysis facility due to differing nephrologist comfort with providing primary care,^14^ as well as by geography with variability in ready availability of a PCP. We classified high-PCP facilities as those in the top tertile of facilities with percentage of patient-years with a primary care visit (eFigure 1 in Supplement 1).

Second, we leveraged the fact that where a patient receives dialysis is almost exclusively a function of their geography without clear links to other factors that may influence outcomes. Differential distance has previously been used as an instrumental variable for studies of stroke,^15^ myocardial infarction,^16,17^ and head trauma.^18^ We used differential distance defined as the difference between the patient’s distance to the nearest high-PCP facility and the patient’s nearest dialysis facility of any type. Given this, if the patient’s nearest dialysis facility was a high-PCP facility, their differential distance would be 0. As differential distance increases, the likelihood of a patient having a PCP decreases (eFigure 2 in Supplement 1). Given that the distribution of differential distance was highly skewed, with a large number of zeros (57 637 patients [31.8%] lived in the same zip code as the nearest high-PCP facility), and a long tail of higher distances, we opted to use a binary variable specification of above or below median differential distance as our instrument. We examined results of a model specifying the instrument as log (differential distance + 1); this produced similar results to our primary binary specification of the instrumental variable (see eTable 2 in Supplement 1). In validating our instrument, the *F* statistic from the first stage model was 4401.25, indicating an association between the instrument and the presence or absence of a PCP.^19^ We also examined whether low- and high-PCP facilities differed in terms of quality of care provided. The mean (SD) dialysis facility compare star ratings for low-PCP facilities was 3.82 (0.97) compared with 3.84 (1.0) for high-PCP facilities. The mean (SD) nurse-to-patient staffing ratio for low-PCP facilities was 0.09 (0.97), compared with 0.10 (0.30) for high-PCP facilities. Given that high- and low-PCP facilities did not differ meaningfully in terms of a global quality of care measure or in a measure of care structure, we felt that differential distance to high-PCP facilities was more likely to be associated with our outcomes through primary care–specific variables.

We included covariates accounting for patient characteristics (age, sex, race and ethnicity, zip code-level median income, county-level education level, dual eligibility for Medicare and Medicaid, receiving a home dialysis modality as of January 1, 2019, zip code in a nonmetropolitan area, and hierarchical condition category [HCC]). Race and ethnicity were categorized as White, Black, Hispanic, and other. The other category included American Indian or Alaska Native, Asian, Native Hawaiian or Pacific Islander, other or multiracial, or unknown, and were collapsed into 1 category due to small sample sizes. The rationale for inclusion of race and ethnicity covariates was as a proxy for socioeconomic, structural, and cultural factors that could affect both the probability of having a PCP and the probability of acute care utilization, thus potentially serving as confounders in the analysis. Dual eligibility for Medicare and Medicaid is used as a marker of low socioeconomic status.^20^ HCCs are a claim-based comorbidity measure developed as a risk adjustment tool and widely used by CMS.^21^ HCCs have been shown to better predict mortality relative to other common indices.^22^ We assigned a nephrologist for each patient based on the unique USRDS physician identifier linked to the first dialysis claim for that patient in the first quarter of 2019.

### Primary and Secondary Outcomes

Our primary outcome of interest was any ED visit between January 1, 2019, and December 31, 2019. Our secondary outcomes of interest were any ED visit not resulting in a hospitalization and any hospitalization between January 1, 2019, and December 31, 2019. These outcomes were determined in a lagged fashion as described in the prior section. These outcomes were chosen because patients receiving dialysis have a higher risk for ED utilization and hospitalization than many other patients with chronic conditions,^23^ and this in turn drives the high cost of care for these patients.^24^

### Statistical Analysis

We compared characteristics between patients with and without a PCP, and those above and below the median differential distance to a high-PCP facility using counts and proportions (Table). We first estimated a multivariable logistic regression model using presence or absence of a PCP at the patient level (hereafter referred to as the patient-level model) to examine the association with our binary outcomes of any ED visit or any ED visit not resulting in admission and any hospitalization, adjusting for the covariates listed previously. We then reported estimated probabilities of each of our outcomes for patients with vs without a PCP. As a second approach, we used an instrumental variable model in a 2-stage residual inclusion estimation framework based on being above or below the median differential distance to a high-PCP facility to examine the association with our outcomes, additionally adjusting for covariates listed previously. For comparability to the output for the patient-level model, we used the margins command to examine the outcomes for patients with an estimated probability of having a PCP of 0% vs 100% by our instrument. All analyses used SAS statistical software, with statistical significance defined as a 2-sided *P* < .05 for trend tests.

**Table.** Characteristics of All Patients in the Cohort When Grouped by Presence or Absence of Primary Care Practitioner or by Those Above or Below Median Differential Distance

Characteristics	Patients, No. (%)					
	Patient had an evaluation and management in 2018			Above median differential distance^a^		
	No	Yes	Std. diff.	Yes	No	Std. diff.
Age, y						
At incidence, mean (SD)	59.4 (14.1)	64.3 (13.7)	0.352	61.9 (14.0)	63.4 (14.1)	0.104
18-39	5990 (9.8)	6162 (5.1)	−0.178	6481 (7.1)	5671 (6.3)	−0.035
40-49	9233 (15.1)	12 079 (10.0)	−0.152	11 355 (12.5)	9957 (11.0)	−0.047
50-59	14 782 (24.1)	23 916 (19.9)	−0.102	19 911 (21.9)	18 787 (20.7)	−0.029
60-69	17 037 (27.8)	34 293 (28.5)	0.016	26 053 (28.7)	25 277 (27.9)	−0.017
70-79	10 171 (16.6)	28 836 (24.0)	0.185	18 626 (20.5)	20 381 (22.5)	0.048
≥80	3999 (6.5)	14 767 (12.3)	0.198	8299 (9.1)	10 467 (11.5)	0.079
Sex						
Female	22 902 (37.4)	55 652 (46.3)	0.182	39 620 (43.6)	38 934 (42.9)	−0.013
Male	38 407 (62.6)	64 559 (53.7)	−0.182	51 250 (56.4)	51 716 (57.1)	0.013
Race and ethnicity						
Black	26 303 (42.9)	45 650 (38.0)	−0.101	35 910 (39.5)	36 043 (39.8)	0.005
Hispanic	9953 (16.2)	17 662 (14.7)	−0.043	14 317 (15.8)	13 298 (14.7)	−0.030
White	31 327 (51.1)	66 334 (55.2)	0.082	49 455 (54.4)	48 206 (53.2)	−0.025
Other race^b^	3679 (6.0)	8227 (6.8)	0.034	5505 (6.1)	6401 (7.1)	0.041
Dual eligibility	31 095 (50.7)	56 805 (47.3)	−0.069	46 103 (50.7)	41 797 (46.1)	−0.093
Receiving home dialysis	7056 (11.5)	11 435 (9.5)	−0.065	9788 (10.8)	8703 (9.6)	−0.039
Education tertile						
1, <9.9% With less than HS (low)	15 021 (24.5)	34 488 (28.7)	0.095	21 332 (23.5)	28 177 (31.1)	0.171
2, 9.9% to <15.6% With less than HS (medium)	26 067 (42.5)	52 608 (43.8)	0.025	38 713 (42.6)	39 962 (44.1)	0.030
3, ≥15.6% With less than HS (high)	20 221 (33.0)	33 115 (27.5)	−0.119	30 825 (33.9)	22 511 (24.8)	−0.201
Median income tertile						
1 (Low)	27 425 (44.7)	46 733 (38.9)	−0.119	41 408 (45.6)	32 750 (36.1)	−0.193
2 (Medium)	18 138 (29.6)	35 038 (29.1)	−0.010	28 521 (31.4)	24 655 (27.2)	−0.092
3 (High)	14 810 (24.2)	36 881 (30.7)	0.147	19 459 (21.4)	32 232 (35.6)	0.317
Nonmetropolitan area	13 686 (22.3)	16 835 (14.0)	−0.217	23 704 (26.1)	6817 (7.5)	−0.513
HCC tertile						
1 (Low)	32 580 (53.1)	30 791 (25.6)	−0.587	33 301 (36.6)	30 070 (33.2)	−0.073
2 (Medium)	18 018 (29.4)	42 531 (35.4)	0.128	30 138 (33.2)	30 411 (33.5)	0.008
3 (High)	10 711 (17.5)	46 889 (39.0)	0.493	27 431 (30.2)	30 169 (33.3)	0.067

Abbreviations: HCC, hierarchical condition category; HS, high school; Std. diff., standardized difference.

^a^ Differential distance equals the distance to closest high–primary care physician (PCP) facility minus the distance to closest facility of any type. High-PCP facility defined as facility in the top tertile of percentage of patient years with a PCP evaluation and management visit.

^b^ Other race includes American Indian or Alaska Native, Asian, Native Hawaiian or Pacific Islander, other or multiracial, or unknown.

We conducted multiple sensitivity analyses to address potential shortcomings in our initial model. First, given that rurality is a known confounder for differential distance as an instrumental variable,^25^ we also conducted a sensitivity analysis limited to facilities in a metropolitan zip code (eTable 3 in Supplement 1). Second, given that the care provided by the nephrologist at the facility (part of facility-level endogeneity) is not accounted for by differential distance, we also conducted a sensitivity analysis with the addition of a nephrologist fixed effect (eTable 4 in Supplement 1). Third, to try and isolate the outcomes of primary care, we identified ED visits with a primary diagnosis that is considered an ambulatory care–sensitive condition (ACSC), as defined by the Agency for Healthcare Research and Quality’s Prevention Quality Indicators.^26^ We specifically used *International Statistical Classification of Diseases and Related Health Problems, Tenth Revision* codes associated with ED visits for diabetes complications (both short and long term), bacterial pneumonia, chronic obstructive pulmonary disease, asthma, or congestive heart failure, which have been used in prior evaluations of ambulatory care sensitive conditions.^27^

## Results

**Figure 2.**
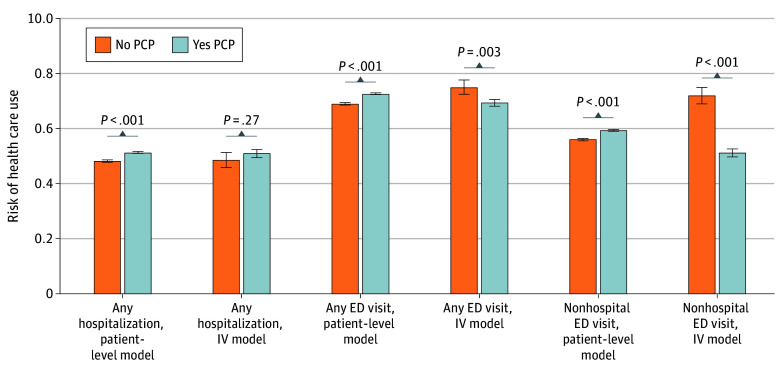
Bar Graph of Estimated Risk of Any Hospitalization, Estimated Risk of Any Emergency Department (ED) Visit, and Estimated Risk of ED Visit Not Resulting in Hospitalization (Non-Hospital ED Visit) Patient-level model separates our cohort by presence or absence of PCP as measured by patient having a primary care evaluation and management code in 2018. IV model separates our cohort by those estimated to have a 100% probability (yes PCP) or 0% probability (no PCP) by our instrument, differential distance to high-PCP facility. IV indicates instrumental variable; PCP, primary care physician.

A total of 181 520 patients (mean [SD] age, 62.6 [14.1]; 102 966 [56.7%] male; 71 953 [39.6%] Black, 27 215 [15.0%] Hispanic, and 97 661 [53.8%] White) with end-stage kidney disease receiving maintenance hemodialysis were included in our analytic cohort. In our patient-level cohort, patients with PCPs were older, suffered a higher burden of comorbidities, were less likely to report Hispanic ethnicity, were less likely to be from a rural area, and were less likely to be receiving a home dialysis modality (Table). In our cohort stratified by patients residing above and below median differential distance to a high-PCP facility, patients were better distributed with respect to multiple variables. The age difference between patients with and without a PCP in the patient-level model was approximately 5 years, shrinking to 1.5 years in the instrumental variable model. The number of comorbidities in the patient-level cohort were unevenly distributed, with 46 889 (39%) of those with a PCP in the highest tertile of number of HCCs compared with only 10 711 (17.5%) of those without a PCP. This distribution improved in the instrumental variable model, with 27 431 (30.2%) of those above median differential distance in the top tertile compared with 30 169** (**33.3%) below the median differential distance. The distribution of patients by median income did not change meaningfully between the patient-level and instrumental variable models. In the patient-level model, 36 881 patients with a PCP (30.7%) were in the highest income tertile relative to 14 810 patients (24.2%) without a PCP in the highest income tertile. In the instrumental variable model, 19 459 patients with above median differential distance (21.4%) were in the highest income tertile compared with 32 232 patients (35.6%) below median differential distance. In the patient-level multivariable logistic regression model, patients with PCPs had higher estimated risk of any hospitalization (51.3%; 95% CI, 51.0%-51.6% vs 48.2%; 95% CI, 47.8%-48.6%; *P* < .001), any ED visit (72.6%; 95% CI, 72.4%-72.9% vs 69.0%; 95% CI, 68.6%-69.3%; *P* < .001), and any ED visit that did not result in hospitalization (59.4%; 95% CI, 59.1%-59.7% vs 56.1%; 95% CI, 55.7%-56.5%; *P* < .001) (Figure 2).

In the instrumental variable model comparing patients estimated to have a 100% probability of having a PCP by our instrument with those estimated to have a 0% probability of having a PCP, there was not a significant difference in the estimated risk of any hospitalization (51.0%; 95% CI, 49.6%-52.4% vs 48.7%; 95% CI, 45.9%-51.4%), but there was a difference in estimated risk of any ED visit (69.4%; 95% CI, 68.1%-70.7% vs 75.0%; 95% CI, 72.5%-77.6%; *P* = .003), particularly in estimated risk of any ED visit not resulting in hospitalization (51.2%; 95% CI, 49.7%-52.7% vs 72.1%; 95% CI, 69.2%-74.9%; *P* < .001). Full 2-stage model results for all outcomes are available in eTables 5 through 7 in Supplement 1.

**Figure 3.**
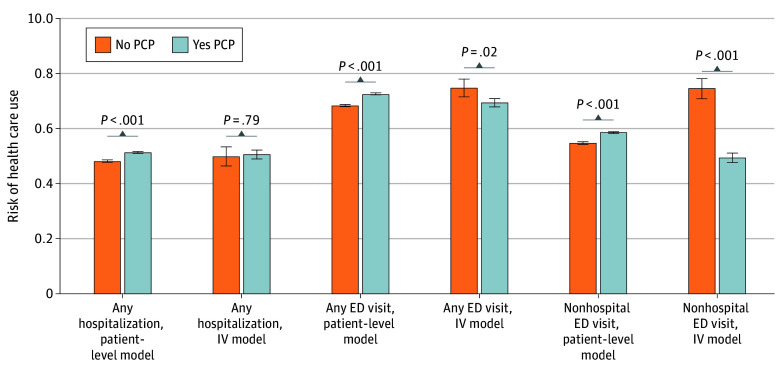
Bar Graph Showing Results of Sensitivity Analysis Restricted to Facilities in Metropolitan Zip Code Estimated risks of any hospitalization, any ED visit, and any ED visit not resulting in hospitalization (nonhospital ED visit). Patient-level model separates our cohort by presence or absence of PCP as measured by patient having a primary care evaluation and management code in 2018. IV separates our cohort by those estimated to have a 100% probability (yes PCP) or 0% probability (no PCP) by our instrument, differential distance to high-PCP facility. ED indicates emergency department; IV, instrumental variable; PCP, primary care physician

Our sensitivity analysis restricted to facilities in metropolitan zip codes (Figure 3) showed similar associations. Our sensitivity analysis with the addition of a nephrologist fixed effect also produced similar associations as in our main model, though the association with reduction in any ED visit was no longer statistically significant (eTable 4 in Supplement 1). Finally, we identified 38 317 patients (21.1%) from our cohort who had at least 1 ACSC-related emergency department visit. Our model showed that patients estimated to have a PCP by our instrument had a 19.8% (95% CI, 18.6%-21.0%) estimated risk of ED visit for an ACSC condition, compared with an estimated risk of 23.7% (95% CI, 21.4%-26.0%; *P* = .03) for patients estimated not to have a PCP by our instrument.

## Discussion

### Findings in Context

In this national retrospective cross-sectional study of patients receiving dialysis, our patient-level model showed an association between the presence of a PCP and increased risk of hospitalization and ED utilization. However, our instrumental variable model showed an association between the estimated presence of a PCP and a reduced risk of any ED visit and any ED visit not resulting in a hospitalization, with a null association with hospitalizations.

Our patient-level model recapitulates findings from prior studies that used similar models.^12^ This difference between the patient-level model and instrumental variable model likely reflects residual confounding in the patient-level model due to patients with PCPs tending to be older and more ill, a factor acknowledged in prior work.^12^ The instrumental variable model potentially better addresses issues of confounding and may provide a more accurate picture of the role of the PCP.

The association of PCPs in our instrumental variable model with ED visits but not hospitalization is in line with prior literature that shows PCP continuity during dialysis transitions was not associated with lower all-cause hospitalization.^29^ This may reflect the challenge of avoiding hospitalization in a particularly comorbid population experiencing an acute illness, limiting the effect PCPs can make incremental to the managing nephrologist.^4^ In contrast, PCPs may be particularly effective in the management of less emergent health concerns in this population given their greater accessibility, experience, and expertise with coordination of care and their generally more holistic view of the patient. Increased after-hours availability of PCPs have previously been linked to lower rates of ED utilization.^30^ PCPs can provide continuity and help coordinate among specialists,^31^ as well as help better manage nonnephrological concerns such as pain management or mental health.^32^ PCPs may also be better suited to provide preventive care, such as vaccination,^33^ reducing ED utilization for preventable illnesses.

### Implications

Emergency department utilization remains a large driver of the cost of care for patients with end-stage kidney disease receiving dialysis.^2^ Current models of care, such as the Kidney Care Choices model, hold large group practices accountable for total cost of care for these patients. Given that primary care involvement may help reduce ED utilization, including partnerships with primary care practices may be one method to reduce total costs of care. Future models of care may benefit from explicitly incentivizing primary care involvement in patients with end-stage kidney disease. This may be part of the reason increased shared savings and lower ED visits were seen with ESCOs.^10^ This will help patients by addressing health issues promptly and ensuring appropriate health maintenance, and also help promote shared savings by reducing costly ED visits. Future analysis directly examining the cost of care and comparing changes in outpatient spending with changes in ED and acute care spending can allow for a more comprehensive analysis of changes in cost of care with PCP involvement in this population.

### Limitations

Our study has certain limitations. One, the use of Medicare Fee for Service claims data excludes patients who are receiving dialysis through private payers or Medicare Advantage, though for the year in question, 2018, a large proportion of the dialysis population was insured through Medicare Fee for Service.^1^ Two, while differential distance accounts for patient-level endogeneity, it does not account for facility-level endogeneity, which may influence the results.^34^ We have attempted to address this concern by showing that high- and low-PCP facilities have similar star quality ratings and nurse-to-patient staffing ratios, as well as conducting a sensitivity analysis with a nephrologist fixed effect, which produced similar findings to our original model. Three, our instrument may serve as a proxy for quality of care, with patients living closer to a dialysis center with primary care involvement also potentially having access to other superior health care services that impact their emergency department utilization. However, sensitivity analyses restricted to metropolitan facilities, which would likely have broadly similar access to health care services within a given market, also produced similar associations as in our main model. Additionally, our instrument estimates the binary presence or absence of a PCP, but does not capture potentially important and nuanced details of PCP involvement such as frequency of visits, continuity of care, or intensity of visits, for which it would be challenging to develop an instrument. Nevertheless, our results are still broadly relevant given that a large proportion of patients with chronic kidney disease receiving dialysis do not see a PCP at all in a given year (see eFigure 2 in Supplement 1).

## Conclusions

In this national retrospective cross-sectional study, an instrumental variable model showed an association between the presence of a PCP for patients with end-stage kidney disease receiving dialysis and a reduced risk of ED visits, particularly ED visits not resulting in a hospitalization. This is distinct from the associations seen in prior cohort studies and a patient-level model, which likely reflects residual confounding present with such approaches. Increasing PCP utilization for patients with end-stage kidney disease receiving dialysis may help reduce ED visits.

## Notes

Supplement 1. **eTable 1.** Healthcare Common Procedure Coding System (HCPCS) Primary Care Service Codes Used to Define Presence or Absence of PCP **eFigure 1.** Caterpillar Plot of Facilities Classified by Percent of Patient Years With a Primary Care E and M Code **eFigure 2.** Percent of Patients With Primary Care E and M Code by Differential Distance Grouping **eTable 2. **Estimated Probabilities of Outcomes for Hospitalization, Any Emergency Department Visit, and Emergency Department Visit Not Resulting in Hospitalization **eTable 3. **Characteristics of Patients in Sensitivity Analysis Excluding Patients Dialyzing in Nonmetropolitan Dialysis Facilities Grouped by Presence or Absence of PCP or by Those Above or Below Median Differential Distance **eTable 4. **Estimated Probabilities for Hospitalization, any Emergency Department Visit, and Emergency Department Visit Not Resulting in Hospitalization Based on Sensitivity Analysis With Model Including Nephrologist Fixed Effect Covariate **eTable 5**. Full 2 Stage Instrumental Variable Results for Outcome of Hospitalization **eTable 6**. Full 2 Stage Instrumental Variable Results for Outcome of Any Emergency Department Visit **eTable 7**. Full 2 Stage Instrumental Variable Results for Outcome of Emergency Department Visit Not Resulting in Hospitalization

Supplement 2. Data Sharing Statement
