# Supplementary material for: Primary Care Involvement and Health Care Utilization Among Patients With End-Stage Kidney Disease
Source: JAMA Netw Open. 2026 Mar 5;9(3):e260807. doi: 10.1001/jamanetworkopen.2026.0807 (PMC12964153; doi:10.1001/jamanetworkopen.2026.0807)
Supplement: Supplement 1. — eTable 1. Healthcare Common Procedure Coding System (HCPCS) Primary Care Service Codes Used to Define Presence or Absence of PCP eFigure 1. Caterpillar Plot of Facilities Classified by Percent of Patient Years With a Primary Care E and M Code eFigure 2. Percent of Patients With Primary Care E and M Code by Differential Distance Grouping eTable 2. Estimated Probabilities of Outcomes for Hospitalization, Any Emergency Department Visit, and Emergency Department Visit Not Resulting in Hospitalization eTable 3. Characteristics of Patients in Sensitivity Analysis Excluding Patients Dialyzing in Nonmetropolitan Dialysis Facilities Grouped by Presence or Absence of PCP or by Those Above or Below Median Differential Distance eTable 4. Estimated Probabilities for Hospitalization, any Emergency Department Visit, and Emergency Department Visit Not Resulting in Hospitalization Based on Sensitivity Analysis With Model Including Nephrologist Fixed Effect Covariate eTable 5. Full 2 Stage Instrumental Variable Results for Outcome of Hospitalization eTable 6. Full 2 Stage Instrumental Variable Results for Outcome of Any Emergency Department Visit eTable 7. Full 2 Stage Instrumental Variable Results for Outcome of Emergency Department Visit Not Resulting in Hospitalization [file jamanetwopen-e260807-s001.pdf]

Supplemental Online Content

Bailoor K, Hirth RA, Guro P, et al. Primary care involvement in the end-stage kidney disease population and health care utilization. *JAMA Netw Open*. 2026;9(3):e260807.
doi:10.1001/jamanetworkopen.2026.0807

eTable 1. Healthcare Common Procedure Coding System (HCPCS) Primary Care Service Codes Used to Define Presence or Absence of PCP

eFigure 1. Caterpillar Plot of Facilities Classified by Percent of Patient Years With a Primary Care E and M Code

eFigure 2. Percent of Patients With Primary Care E and M Code by Differential Distance Grouping

eTable 2. Estimated Probabilities of Outcomes for Hospitalization, Any Emergency Department Visit, and Emergency Department Visit Not Resulting in Hospitalization

eTable 3. Characteristics of Patients in Sensitivity Analysis Excluding Patients Dialyzing in Nonmetropolitan Dialysis Facilities Grouped by Presence or Absence of PCP or by Those Above or Below Median Differential Distance

eTable 4. Estimated Probabilities for Hospitalization, any Emergency Department Visit, and Emergency Department Visit Not Resulting in Hospitalization Based on Sensitivity Analysis With Model Including Nephrologist Fixed Effect Covariate

eTable 5. Full 2 Stage Instrumental Variable Results for Outcome of Hospitalization

eTable 6. Full 2 Stage Instrumental Variable Results for Outcome of Any Emergency Department Visit

eTable 7. Full 2 Stage Instrumental Variable Results for Outcome of Emergency Department Visit Not Resulting in Hospitalization

This supplemental material has been provided by the authors to give readers additional information about their work.

© 2026 Bailoor K et al. *JAMA Network Open*.

eTable 1. Healthcare Common Procedure Coding System (HCPCS) Primary Care Service Codes Used to Define Presence or Absence of PCP

Considered patient to have PCP if a provider with specialty codes 01 (general practice), 08 (family practice), 11 (internal medicine), 37 (pediatric medicine), and 38 (geriatric medicine) submitted a claim with one of these codes between 01/01/2018 and 12/31/2018.

**HCPCS Codes**	**Brief Description**
99201-99205	New patient, office, or other outpatient visit
99211-99215	Established patient, office, or other outpatient visit
99304-99306	New patient, nursing facility care
99307-99310	Established patient, nursing facility care
99315-99316	Established patient, discharge day management service
99318	Established patient, other nursing facility service
99324-99328	New patient, domiciliary or rest home visit
99339-99340	Established patient, physician supervision of patient (patient not present) in home, domiciliary, or rest home
99341-99345	New patient, home visit
99347-99350	Established patient, home visit
G0402	Initial Medicare visit
G0438	Annual wellness visit, initial
G0429	Annual wellness visit, subsequent
G0463	Hospital outpatient clinic visit

© 2026 Bailoor K et al. *JAMA Network Open*.

eFigure 1. Caterpillar Plot of Facilities Classified by Percent of Patient Years With a Primary Care E and M Code
Excluding facilities with less than 5 patient years of data. Each dot represents the average for 5 facilities. Solid line represents overall average at 62%.

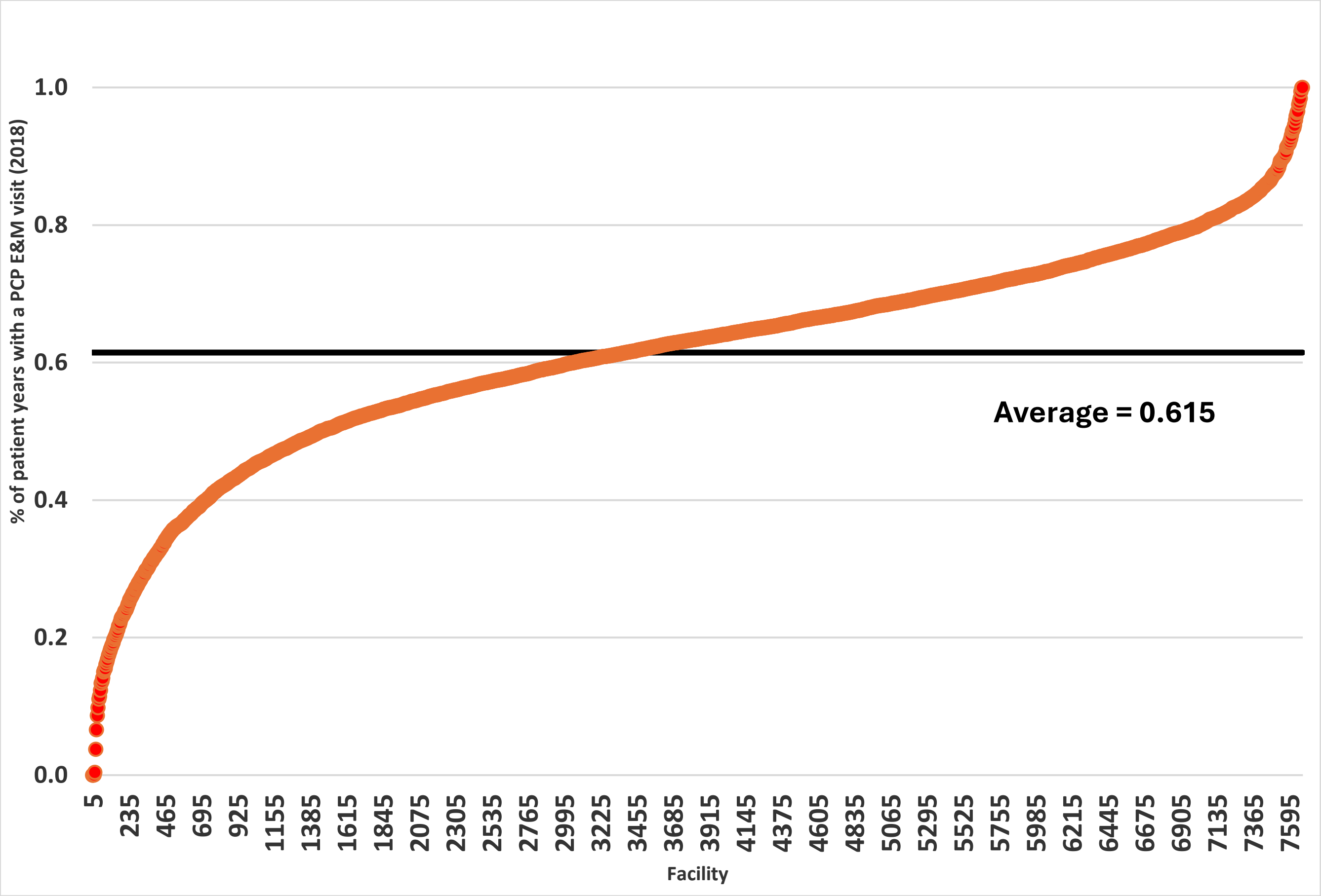

Abbreviations: E&M, evaluation and management; PCP, primary care physician.

© 2026 Bailoor K et al. *JAMA Network Open*.

eFigure 2. Percent of Patients With Primary Care E and M Code by Differential Distance Grouping

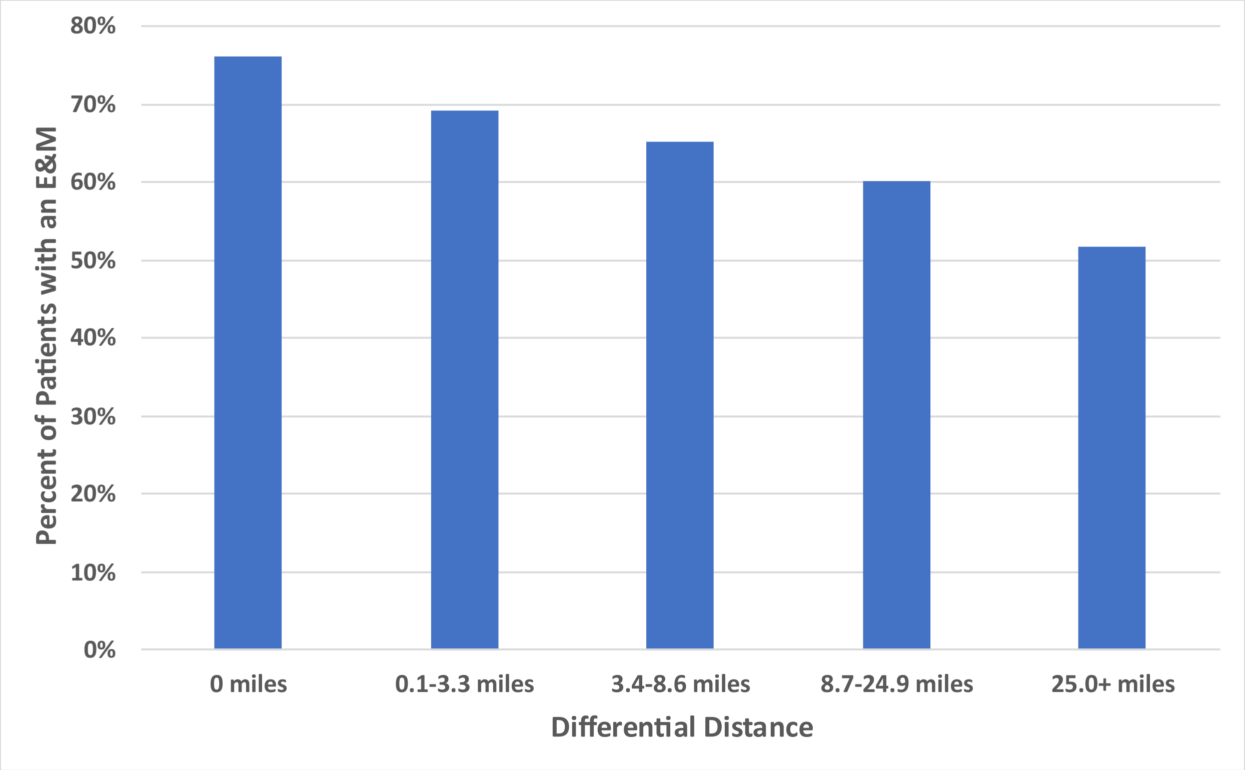

Abbreviations: E&M, evaluation and management.

© 2026 Bailoor K et al. *JAMA Network Open*.

eTable 2. Estimated Probabilities of Outcomes for Hospitalization, Any Emergency Department Visit, and Emergency Department Visit Not Resulting in Hospitalization

When using log (differential distance +1) as instrument with same co-variates as main model.

Outcome	*Estimated Risk for Patients Predicted To Have PCP*	*95% Confidence Interval*	*Estimated Risk for Patients Not to Have PCP*	*95% Confidence Interval*	*p-value*
ER Visit Without Hospitalization	52.6%	51.4%-53.8%	69.2%	66.9%-71.6%	<0.001
Any ER Visit	70.1%	69.0%-71.2%	73.7%	71.6%-75.8%	0.025
Hospitalization	51.8%	50.6%-53.0%	47.2%	44.9%-49.5%	0.011

Abbreviations: ER, emergency room; PCP, primary care physician.

© 2026 Bailoor K et al. *JAMA Network Open*.

eTable 3. Characteristics of Patients in Sensitivity Analysis Excluding Patients Dialyzing in Nonmetropolitan Dialysis Facilities Grouped by Presence or Absence of PCP or by Those Above or Below Median Differential Distance

Differential distance equals the distance to closest high PCP facility minus the distance to closest facility of any type. High PCP facility defined as facility in the top tertile of % of patient years with a PCP E&M visit.

	Exclude Non-Metropolitan Dialysis Facilities						
	Patient had an E&M in 2018				Above Median Differential Distance[a]		
Patient Characteristics	No N (%)	Yes N (%)	Std. Diff.		Yes N (%)	No N (%)	Std. Diff.
Age at Incidence, mean (SD)	59.0 (14.1)	64.3 (13.7)	0.379		61.7 (14.0)	63.4 (14.1)	0.116
Age 18-39	4796 (10.1)	5304 (5.1)	-0.187		4860 (7.2)	5240 (6.3)	-0.039
Age 40-49	7382 (15.5)	10295 (10.0)	-0.167		8475 (12.6)	9202 (11.0)	-0.051
Age 50-59	11695 (24.6)	20595 (19.9)	-0.112		14891 (22.2)	17399 (20.8)	-0.035
Age 60-69	13114 (27.5)	29489 (28.5)	0.022		19215 (28.6)	23388 (27.9)	-0.016
Age 70-79	7578 (15.9)	24736 (23.9)	0.202		13572 (20.2)	18742 (22.4)	0.053
Age 80+	2985 (6.3)	12820 (12.4)	0.212		6037 (9.0)	9768 (11.7)	0.088
Female	17281 (36.3)	47598 (46.0)	0.199		28973 (43.1)	35906 (42.8)	-0.006
Male	30342 (63.7)	55778 (54.0)	-0.199		38193 (56.9)	47927 (57.2)	0.006
Black	21272 (44.7)	40395 (39.1)	-0.114		27212 (40.5)	34455 (41.1)	0.012
White	23287 (48.9)	56002 (54.2)	0.106		35600 (53.0)	43689 (52.1)	-0.018
Other Race	3064 (6.4)	6979 (6.8)	0.013		4354 (6.5)	5689 (6.8)	0.012
Hispanic Ethnicity	8654 (18.2)	16417 (15.9)	-0.061		12152 (18.1)	12919 (15.4)	-0.072
Dual Eligibility	24007 (50.4)	48390 (46.8)	-0.072		33895 (50.5)	38502 (45.9)	-0.091
On Home Dialysis	5275 (11.1)	9614 (9.3)	-0.059		6947 (10.3)	7942 (9.5)	-0.029
Less Than HS Education Tertile 1 Low	12971 (27.2)	31599 (30.6)	0.074		17980 (26.8)	26590 (31.7)	0.109
Less Than HS Education Tertile 2 Medium	21927 (46.0)	47210 (45.7)	-0.008		31413 (46.8)	37724 (45.0)	-0.036
Less Than HS Education Tertile 3 High	12725 (26.7)	24567 (23.8)	-0.068		17773 (26.5)	19519 (23.3)	-0.074
Median Income Tertile 1 Low	18405 (38.6)	36110 (34.9)	-0.077		25598 (38.1)	28917 (34.5)	-0.075
Median Income Tertile 2 Medium	14314 (30.1)	30122 (29.1)	-0.020		22017 (32.8)	22419 (26.7)	-0.132

© 2026 Bailoor K et al. *JAMA Network Open*.

Median Income Tertile 3 High	14235 (29.9)	35941 (34.8)	0.104		18580 (27.7)	31596 (37.7)	0.215
Non-Metropolitan Area							
HCC Tertile 1 Low	25810 (54.2)	26321 (25.5)	-0.614		24382 (36.3)	27749 (33.1)	-0.067
HCC Tertile 2 Medium	13801 (29.0)	36620 (35.4)	0.138		22223 (33.1)	28198 (33.6)	0.012
HCC Tertile 3 High	8012 (16.8)	40435 (39.1)	0.513		20561 (30.6)	27886 (33.3)	0.057

[a]Differential Distance = Distance to closest high PCP facility - distance to closest facility. High PCP facility = facility in the top tertile of % of patient years with a PCP E&M visit.

Abbreviations: E&M, evaluation and management; ER, emergency room; HCC, Hierarchical Condition Category; HS, high school; N, number; PCP, primary care physician; SD, standard deviation; Std. diff, standardized difference.

© 2026 Bailoor K et al. *JAMA Network Open*.

eTable 4. Estimated Probabilities for Hospitalization, any Emergency Department Visit, and Emergency Department Visit Not Resulting in Hospitalization Based on Sensitivity Analysis With Model Including Nephrologist Fixed Effect Covariate

**Predicted Probabilities of outcomes from model with nephrologist fixed effect**					
Outcome	*Estimated Risk for Patients Below Median Differential Distance*	*95% Confidence Interval*	*Estimated Risk for Patients Above Median Differential Distance*	*95% Confidence Interval*	*p-value*
ER Visit without Admission	54.1%	49.6%-58.7%	69.4%	60.0%-78.7%	0.032
Any ER Visit	69.0%	64.5%-73.1%	78.8%	70.4%-87.3%	0.125
Hospitalization	52.8%	48.3%-57.3%	47.2%	38.0%-56.5%	0.424

Abbreviations: ER, emergency room.

© 2026 Bailoor K et al. *JAMA Network Open*.

eTable 5. Full 2 Stage Instrumental Variable Results for Outcome of Hospitalization

**First-stage regressions: Independent Variable Primary Care E&M Code in 2018**					
Dependent Variable	*Coefficient*	*Standard Error*	*p-value*	*95% CI Lower Bound*	*95% CI Upper Bound*
Age (per 1 year increase)	0.004	<0.001	<0.001	0.003	0.004
Female (relative to male)	0.059	0.002	<0.001	0.055	0.064
Black Race (relative to White)	-0.34	0.002	<0.001	-0.039	-0.029
Other Race (relative to White)	0.020	0.004	<0.001	0.012	0.029
Hispanic Ethnicity (relative to non-Hispanic ethnicity)	-0.024	0.003	<0.001	-0.031	-0.018
Dual-eligible for Medicaid/Medicare	-0.003	0.002	0.114	-0.008	0.001
On Home Dialysis	0.017	0.003	<0.001	0.010	0.024
Less Than HS Education Tertile 2 Medium (relative to Tertile 1 Low)	-0.006	0.002	0.019	-0.011	-0.001
Less Than HS Education Tertile 3 High (relative to Tertile 1 Low)	-0.017	0.003	<0.001	-0.023	-0.011
Median Income Tertile 2 Medium (relative to Tertile 1 Low)	0.003	0.002	0.255	-0.002	0.008
Median Income Tertile 3 High (relative to Tertile 1 Low)	0.012	0.003	<0.001	0.007	0.018
Non-metropolitan Area (relative to metropolitan)	-0.087	0.003	<0.001	-0.093	-0.081
Total HCCs Tertile 2 Medium (relative to Total HCCs Tertile 1 Low)	0.200	0.002	<0.001	0.194	0.204
Total HCCs Tertile 3 High (relative to Total HCCs Tertile 1 Low)	0.305	0.003	<0.001	0.300	0.310
Below Median Differential Distance (relative to Above Median Differential Distance)	0.110	0.002	<0.001	0.106	0.114
_cons	0.194	0.006	<0.001	0.182	0.207
**Instrumental-variables 2SLS Regression: Independent Variable Hospitalization**					

© 2026 Bailoor K et al. *JAMA Network Open*.

Dependent Variable	*Coefficient*	*Standard Error*	*p-value*	*95% CI Lower Bound*	*95% CI Upper Bound*
Had Primary Care E&M Code in 2018	0.023	0.021	0.270	-0.018	0.065
Age (per 1 year increase)	0.001	0.001	0.332	-0.001	0.001
Female (relative to male)	0.036	0.003	<0.001	0.030	0.041
Black Race (relative to White)	-0.029	0.002	<0.001	-0.035	-0.024
Other Race (relative to White)	-0.027	0.005	<0.001	-0.036	-0.017
Hispanic Ethnicity (relative to non-Hispanic ethnicity)	-0.013	0.004	<0.001	-0.020	-0.006
Dual-eligible for Medicaid/Medicare	0.036	0.002	<0.001	0.031	0.040
On Home Dialysis	0.067	0.004	<0.001	0.060	0.075
Less Than HS Education Tertile 2 Medium (relative to Tertile 1 Low)	0.007	0.003	0.019	0.001	0.012
Less Than HS Education Tertile 3 High (relative to Tertile 1 Low)	-0.001	0.003	0.836	-0.007	0.006
Median Income Tertile 2 Medium (relative to Tertile 1 Low)	-0.010	0.003	<0.001	-0.016	-0.005
Median Income Tertile 3 High (relative to Tertile 1 Low)	-0.025	0.003	<0.001	-0.031	-0.019
Non-metropolitan Area (relative to metropolitan)	-0.015	0.004	<0.001	-0.024	-0.007
Total HCCs Tertile 2 Medium (relative to Total HCCs Tertile 1 Low)	0.157	0.005	<0.001	0.147	0.167
Total HCCs Tertile 3 High (relative to Total HCCs Tertile 1 Low)	0.338	0.007	<0.001	0.324	0.352
_cons	0.305	0.009	<0.001	0.288	0.322

Abbreviations: CI, confidence interval; E&M, evaluation and management; HCC, Hierarchical Condition Category; HS, high school.

© 2026 Bailoor K et al. *JAMA Network Open*.

eTable 6. Full 2 Stage Instrumental Variable Results for Outcome of Any Emergency Department Visit

**First-stage regressions: Independent Variable Primary Care E&M Code in 2018**					
Dependent Variable	*Coefficient*	*Standard Error*	*p-value*	*95% CI Lower Bound*	*95% CI Upper Bound*
Age (per 1 year increase)	0.004	<0.001	<0.001	0.003	0.004
Female (relative to male)	0.059	0.002	<0.001	0.055	0.064
Black Race (relative to White)	-0.34	0.002	<0.001	-0.039	-0.029
Other Race (relative to White)	0.020	0.004	<0.001	0.012	0.029
Hispanic Ethnicity (relative to non-Hispanic ethnicity)	-0.024	0.003	<0.001	-0.031	-0.018
Dual-eligible for Medicaid/Medicare	-0.003	0.002	0.114	-0.008	0.001
On Home Dialysis	0.017	0.003	<0.001	0.010	0.024
Less Than HS Education Tertile 2 Medium (relative to Tertile 1 Low)	-0.006	0.002	0.019	-0.011	-0.001
Less Than HS Education Tertile 3 High (relative to Tertile 1 Low)	-0.017	0.003	<0.001	-0.023	-0.011
Median Income Tertile 2 Medium (relative to Tertile 1 Low)	0.003	0.002	0.255	-0.002	0.008
Median Income Tertile 3 High (relative to Tertile 1 Low)	0.012	0.003	<0.001	0.007	0.018
Non-metropolitan Area (relative to metropolitan)	-0.087	0.003	<0.001	-0.093	-0.081
Total HCCs Tertile 2 Medium (relative to Total HCCs Tertile 1 Low)	0.200	0.002	<0.001	0.194	0.204
Total HCCs Tertile 3 High (relative to Total HCCs Tertile 1 Low)	0.305	0.003	<0.001	0.300	0.310
Below Median Differential Distance (relative to Above Median Differential Distance)	0.110	0.002	<0.001	0.106	0.114
_cons	0.194	0.006	<0.001	0.182	0.207
**Instrumental-variables 2SLS Regression: Independent Variable Any ER Visit**					

© 2026 Bailoor K et al. *JAMA Network Open*.

Dependent Variable	*Coefficient*	*Standard Error*	*p-value*	*95% CI Lower Bound*	*95% CI Upper Bound*
Had Primary Care E&M Code in 2018	-0.057	0.019	0.003	-0.095	-0.019
Age (per 1 year increase)	-0.001	<0.001	0.905	-0.001	0.001
Female (relative to male)	0.059	0.002	<0.001	0.055	0.064
Black Race (relative to White)	0.009	0.002	<0.001	0.004	0.014
Other Race (relative to White)	-0.058	0.004	<0.001	-0.066	-0.049
Hispanic Ethnicity (relative to non-Hispanic ethnicity)	0.004	0.003	0.225	-0.002	0.011
Dual-eligible for Medicaid/Medicare	0.066	0.002	<0.001	0.061	0.070
On Home Dialysis	0.024	0.003	<0.001	0.017	0.030
Less Than HS Education Tertile 2 Medium (relative to Tertile 1 Low)	-0.005	0.003	0.053	-0.010	0.001
Less Than HS Education Tertile 3 High (relative to Tertile 1 Low)	-0.031	0.003	<0.001	-0.037	-0.025
Median Income Tertile 2 Medium (relative to Tertile 1 Low)	-0.004	0.003	0.108	-0.009	0.001
Median Income Tertile 3 High (relative to Tertile 1 Low)	-0.011	0.003	<0.001	-0.016	-0.005
Non-metropolitan Area (relative to metropolitan)	0.004	0.004	0.233	-0.003	-0.005
Total HCCs Tertile 2 Medium (relative to Total HCCs Tertile 1 Low)	0.166	0.005	<0.001	0.157	0.175
Total HCCs Tertile 3 High (relative to Total HCCs Tertile 1 Low)	0.292	0.006	<0.001	0.279	0.305
_cons	0.558	0.008	<0.001	0.542	0.573

Abbreviations: CI, confidence interval; E&M, evaluation and management; ER, emergency room; HCC, Hierarchical Condition Category; HS, high school.

© 2026 Bailoor K et al. *JAMA Network Open*.

eTable 7. Full 2 Stage Instrumental Variable Results for Outcome of Emergency Department Visit Not Resulting in Hospitalization

**First-stage regressions: Independent Variable Primary Care E&M Code in 2018**					
Dependent Variable	*Coefficient*	*Standard Error*	*p-value*	*95% CI Lower Bound*	*95% CI Upper Bound*
Age (per 1 year increase)	0.004	<0.001	<0.001	0.003	0.004
Female (relative to male)	0.059	0.002	<0.001	0.055	0.064
Black Race (relative to White)	-0.34	0.002	<0.001	-0.039	-0.029
Other Race (relative to White)	0.020	0.004	<0.001	0.012	0.029
Hispanic Ethnicity (relative to non-Hispanic ethnicity)	-0.024	0.003	<0.001	-0.031	-0.018
Dual-eligible for Medicaid/Medicare	-0.003	0.002	0.114	-0.008	0.001
On Home Dialysis	0.017	0.003	<0.001	0.010	0.024
Less Than HS Education Tertile 2 Medium (relative to Tertile 1 Low)	-0.006	0.002	0.019	-0.011	-0.001
Less Than HS Education Tertile 3 High (relative to Tertile 1 Low)	-0.017	0.003	<0.001	-0.023	-0.011
Median Income Tertile 2 Medium (relative to Tertile 1 Low)	0.003	0.002	0.255	-0.002	0.008
Median Income Tertile 3 High (relative to Tertile 1 Low)	0.012	0.003	<0.001	0.007	0.018
Non-metropolitan Area (relative to metropolitan)	-0.087	0.003	<0.001	-0.093	-0.081
Total HCCs Tertile 2 Medium (relative to Total HCCs Tertile 1 Low)	0.200	0.002	<0.001	0.194	0.204
Total HCCs Tertile 3 High (relative to Total HCCs Tertile 1 Low)	0.305	0.003	<0.001	0.300	0.310
Below Median Differential Distance (relative to Above Median Differential Distance)	0.110	0.002	<0.001	0.106	0.114
_cons	0.194	0.006	<0.001	0.182	0.207
**Instrumental-variables 2SLS Regression: Independent Variable ER Visit Not Resulting in Hospitalization**					

© 2026 Bailoor K et al. *JAMA Network Open*.

Dependent Variable	*Coefficient*	*Standard Error*	*p-value*	*95% CI Lower Bound*	*95% CI Upper Bound*
Had Primary Care E&M Code in 2018	-0.209	0.022	<0.001	-0.251	-0.166
Age (per 1 year increase)	-0.001	0.001	<0.001	-0.001	-0.001
Female (relative to male)	0.078	0.003	<0.001	0.072	0.083
Black Race (relative to White)	0.022	0.005	<0.001	0.017	0.028
Other Race (relative to White)	-0.069	0.005	<0.001	-0.079	-0.059
Hispanic Ethnicity (relative to non-Hispanic ethnicity)	0.008	0.004	0.030	0.001	0.015
Dual-eligible for Medicaid/Medicare	0.074	0.003	<0.001	0.069	0.079
On Home Dialysis	-0.003	0.004	0.452	-0.010	0.005
Less Than HS Education Tertile 2 Medium (relative to Tertile 1 Low)	-0.013	0.003	<0.001	-0.019	-0.007
Less Than HS Education Tertile 3 High (relative to Tertile 1 Low)	-0.047	0.003	<0.001	-0.054	-0.041
Median Income Tertile 2 Medium (relative to Tertile 1 Low)	-0.001	0.003	0.760	-0.006	0.005
Median Income Tertile 3 High (relative to Tertile 1 Low)	-0.021	0.003	<0.001	-0.027	-0.015
Non-metropolitan Area (relative to metropolitan)	0.030	0.004	<0.001	0.022	0.039
Total HCCs Tertile 2 Medium (relative to Total HCCs Tertile 1 Low)	0.176	0.005	<0.001	0.295	0.323
Total HCCs Tertile 3 High (relative to Total HCCs Tertile 1 Low)	0.309	0.007	<0.001	0.295	0.323
_cons	0.543	0.009	<0.001	0.526	0.560

Abbreviations: CI, confidence interval; E&M, evaluation and management; ER, emergency room; HCC, Hierarchical Condition Category; HS, high school.

© 2026 Bailoor K et al. *JAMA Network Open*.
