# Supplementary material for: Primary Care Involvement and Health Care Utilization Among Patients With End-Stage Kidney Disease
Source: JAMA Netw Open. 2026 Mar 5;9(3):e260807. doi: 10.1001/jamanetworkopen.2026.0807 (PMC12964153; doi:10.1001/jamanetworkopen.2026.0807)
Supplement: Supplement 2. — Data Sharing Statement [file jamanetwopen-e260807-s002.pdf]

Data Sharing Statement

Bailoor. Primary Care Involvement in the End-Stage Kidney Disease Population and Health Care Utilization. *JAMA Netw Open*. Published March 05, 2026. doi:10.1001/jamanetworkopen.2026.0807

Data
Data available: No

Additional Information
Explanation for why data not available: Under data use agreement with United States Renal Data Systems, we are not at liberty to share patient data
